# Magnetocaloric effect in a high-spin ferromagnetic molecular cluster

**DOI:** 10.3389/fchem.2024.1494609

**Published:** 2024-10-31

**Authors:** Eleftheria Agapaki, Emmanouil K. Charkiolakis, Gary S. Nichol, David Gracia, Marco Evangelisti, Euan K. Brechin

**Affiliations:** ^1^ EaStCHEM School of Chemistry, The University of Edinburgh, Edinburgh, Scotland, United Kingdom; ^2^ Instituto de Nanociencia y Materiales de Aragón (INMA), CSIC & Universidad de Zaragoza, Zaragoza, Spain

**Keywords:** cluster compounds, magnetic properties, ferromagnetism, magnetocaloric effect, manganese, manganese

## Abstract

The reaction of MnCl_2_·4H_2_O with HL ((1-methyl-1H-imidazol-2-yl)methanol) and pdH_2_ (1, 3 propanediol) in a basic MeCN solution results in the formation of a mixed-valence [Mn_20_] cationic cluster and two [Mn^II^Cl_4_] counter anions. The metallic skeleton of the cluster describes two geometrically equivalent mixed-valent, linked [Mn^III^_6_Mn^II^_4_] supertetrahedra in which nearest-neighbor metal ions have a different oxidation state. Magnetic susceptibility, magnetization data and heat capacity measurements support evidence of predominant ferromagnetic correlations, leading to a *s* = 22 spin ground state for the [Mn^III^_6_Mn^II^_4_] supertetrahedra, which are pair-linked by a weak antiferromagnetic coupling. The properties are discussed in the context of the magnetocaloric effect and the potential application of this compound in cryogenic refrigeration.

## 1 Introduction

As early as the 1990s, seminal publications promoted the use of ferromagnetic particles for magnetic refrigeration (McMichael et al., 1992; Shull, 1993; Bennett et al., 1994). Clustering spin moments into noninteracting particles results in a net magnetic moment per particle. An applied magnetic field can align the large magnetic moments of ferromagnetic particles more easily than a magnetic domain of similar size in the bulk equivalent paramagnetic material, at least for certain temperatures and particle sizes. In a magnetocaloric material, the change of the applied magnetic field induces a change in the material’s magnetic entropy (Δ*S*_m_) and adiabatic temperature (Δ*T*_ad_). The magnetocaloric effect (MCE) can therefore be substantial, and enhanced, in ferromagnetic particles. However, interparticle interactions, size distributions, and the presence of non-active solvent, are all ingredients that negatively affect their performance in terms of the MCE.

Magnetic molecular clusters inherit the advantages of ferromagnetic particles and are in many ways superior because of ideal monodispersity in size, shape and magnetic moment. In addition, their molecular nature opens avenues for fine tuning properties (Evangelisti et al., 2011; Sharples et al., 2014; Tziotzi et al., 2023; Zhai et al., 2024). This last point is crucial for improving their MCE (Evangelisti and Brechin, 2010). Sought-after molecular clusters are those with a large spin ground state and a small magnetic anisotropy, because of their easier polarization by the applied magnetic field. At first, the archetypal “single-molecule magnets” such as [Mn_12_] and [Fe_8_] were proposed for magnetic refrigeration (Torres et al., 2000; Zhang et al., 2001; Spichkin et al., 2001), but their huge anisotropies limit their applicability as refrigerants despite the relatively large *s* = 10 spin ground state. The search for isotropic molecular clusters led to heterometallic Cr-based wheels, whose limitations are in the small value of their spin ground state (Affronte et al., 2004). The first high-spin and low-anisotropy molecular cluster was the highly symmetric supertetrahedron [Mn_10_O_4_Br_4_(amp)_6_(ampH_2_)_3_(HampH_2_)]Br_3_ (ampdH_2_ = 2-amino-2-methyl-1,3-propanediol), a mixed-valent ([Mn^III^_6_Mn^II^_4_]) ferromagnetic cluster with a remarkable *s* = 22 ground state displaying negligible anisotropy (Manoli et al., 2007; Manoli et al., 2008). Most of the focus has shifted since then into gadolinium-containing molecular clusters, first in the form of mixed 3d-4f clusters (Karotsis et al., 2009), then as purely gadolinium-based clusters (Evangelisti et al., 2011). Gadolinium is nowadays considered as the standard element for any new molecular cluster for magnetic refrigeration, the advantage residing in its large *s* = 7/2 moment, zero orbital angular momentum and weak magnetic correlations that, together, facilitate record-high MCE values (Konieczny et al., 2022; Tziotzi et al., 2023). However, for commercial uptake, magnetocaloric materials should be made from safe, inexpensive, and abundant elements (Chaudhary et al., 2019). Since gadolinium is treated as critical because of the concerns surrounding its supply (Zhao et al., 2023), it could be replaced, for instance, by the earth abundant high spin, Fe^III^ and Mn^II^ ions without significantly deteriorating the MCE due to a not much smaller *s* = 5/2. Here, we show that the use of 1,3-propanediol in combination with the N,O-chelate (1-methyl-1H-imidazol-2-yl)methanol (HL) can be used to isolate the cluster [Mn^III^_12_Mn^II^_8_O_8_(L)_16_(HL)_2_(pd)_4_(pdH_2_)Cl_8_][Mn^II^Cl_4_]_2_·3MeCN·9C_2_H_6_O (**1**·3MeCN·9C_2_H_6_O), which is structurally related to [Mn_10_O_4_Br_4_(amp)_6_(ampH_2_)_3_(HampH_2_)]Br_3_ (Manoli et al., 2007). Compound **1** contains two analogous [Mn^III^_6_Mn^II^_4_] supertetrahedra carrying a *s* = 22 ground state, but on this occasion, they are covalently linked into a [Mn_10_]_2_ dimer through a single pdH_2_ bridge. In comparison with [Mn_10_O_4_Br_4_(amp)_6_(ampH_2_)_3_(HampH_2_)]Br_3_ (Manoli et al., 2007), the structure of **1** is considerably lighter, which ultimately promotes a larger MCE, favored by the larger weight of magnetic elements with respect to nonmagnetic ones, which act passively (Lorusso et al., 2013; Tziotzi et al., 2023).

## 2 Experimental methods

### 2.1 Synthesis

MnCl_2_·4H_2_O (198 mg, 1 mmol), HL (112 mg, 1 mmol), pdH_2_ (72 μL, 1 mmol) and NEt_3_ (420 μL, 3 mmol) were dissolved in MeCN (15 mL) and stirred for 1 h. The solution was then filtered and diffused with acetone. Brown crystals of **1** were obtained after 2 days. Elemental analysis (%) calculated for Mn_22_O_45_N_56_C_133_H_216_Cl_16_: C, 31.35; H, 4.27; N, 15.39. Found: C, 31.42; H, 4.38; N, 15.27. Yield ≤40%.

### 2.2 Single-crystal X-ray diffraction

A suitable brown, blade-shaped crystal of **1**·3MeCN·9C_2_H_6_O with dimensions 0.18 × 0.07 × 0.04 mm^3^ was selected and mounted on a MITIGEN holder in perfluoroether oil on a Rigaku Oxford Diffraction SuperNova diffractometer. The crystal was kept at a steady *T* = 100.00 K during data collection. The structure was solved with the ShelXT (Sheldrick, 2015a) solution program using dual methods and by using Olex2 1.5-beta (Dolomanov et al., 2009) as the graphical interface. The model was refined with ShelXL 2018/3 (Sheldrick, 2015b) using full matrix least squares minimization on *F* ^2^. Crystal Data. C_133.4_H_216.41_Cl_16_Mn_22_N_36.39_O_45.32_, *M*_*r*_ = 4,831.09, triclinic, *P*-1 (No. 2), *a* = 14.7918(17) Å, *b* = 18.444(2) Å, *c* = 19.275(2) Å, *α* = 82.653(5)^°^, *β* = 69.696(5)^°^, *γ* = 80.063(5)^°^, *V* = 4,844.7(10) Å^3^, *T* = 100.00 K, *Z* = 1, *Z'* = 0.5, *m*(MoK_*a*_) = 1.675, 279539 reflections measured, 26124 unique (*R*_int_ = 0.0580) which were used in all calculations. The final *wR*_*2*_ was 0.1620 (all data) and *R*_*1*_ was 0.0558 (I ≥ 2σ(I)). CCDC = 2342033.

### 2.3 Physical properties measurements

Magnetization and magnetic susceptibility data were collected on a freshly prepared polycrystalline powder of **1** on a Quantum Design MPMS3 SQUID magnetometer, equipped with a 7 T magnet in the temperature range 2–300 K. Diamagnetic corrections were applied to the observed paramagnetic susceptibilities using Pascal’s constants. Heat capacity measurements were carried out using a Quantum Design PPMS, equipped with a ^3^He option and a 9 T magnet in the temperature range 0.3–30 K. The polycrystalline sample of **1** was in the form of a thin pressed pellet (ca. 1 mg), thermalized by ca. 0.2 mg of Apiezon N grease, whose contribution was subtracted by using a phenomenological expression.

## 3 Results and discussion

**FIGURE 1:**
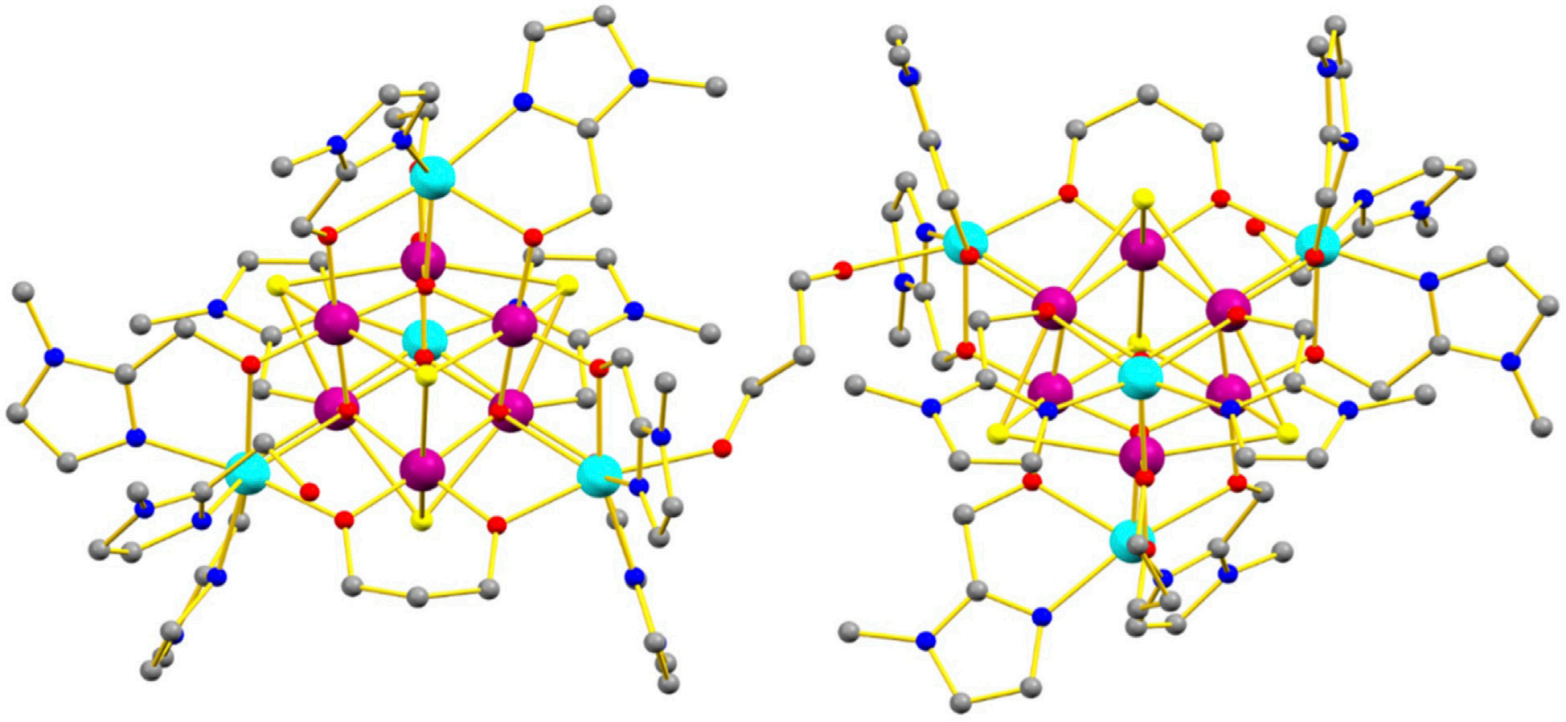
Molecular structure of **1**. Color code: Mn^III^ = purple, Mn^II^ = cyan, O = red, N = blue, C = grey, Cl = yellow. Solvent molecules, anions and H atoms omitted for clarity.

The reaction of MnCl_2_·4H_2_O with HL ((1-methyl-1H-imidazol-2-yl)methanol) and pdH_2_ (1,3-propanediol) in a basic MeCN solution results in the formation of the mixed-valence compound [Mn^III^_12_Mn^II^_8_O_8_(L)_16_(HL)_2_(pd)_4_(pdH_2_)Cl_8_][Mn^II^Cl_4_]_2_·3MeCN·9C_2_H_6_O in (**1**), upon diffusion of acetone into the mother liquor (Figure 1). Compound **1** crystallizes in the triclinic space group *P*-1 with the asymmetric unit comprising half of the formula.

**FIGURE 2:**
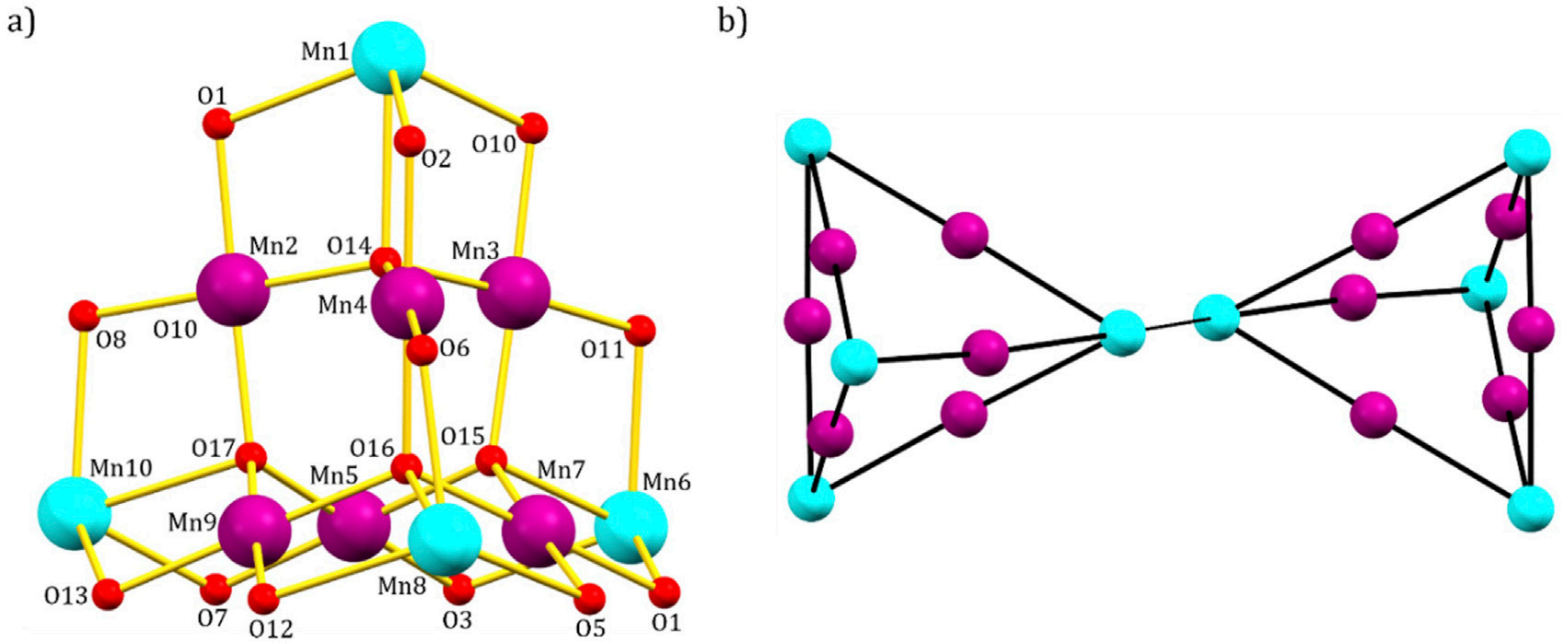
**(A)** Metal-oxygen core; **(B)** metallic skeleton of **1**. Color code: Mn^III^ = purple, Mn^II^ = cyan, O = red.

The metallic skeleton of **1** (Figure 2) describes two geometrically equivalent mixed-valent [Mn^III^_6_Mn^II^_4_] supertetrahedra, bridged via a protonated pdH_2_ ligand through Mn_8_ (and symmetry equivalent (s. e.), Mn^II^-Mn^II^). Nearest neighbors within each supertetrahedron have a different oxidation state (Mn^II^ = Mn1, Mn6, Mn8, Mn10; Mn^III^ = Mn2, Mn3, Mn4, Mn5, Mn7, Mn9).

**SCHEME 1:**
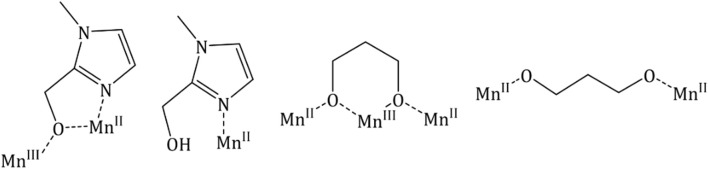
Bridging modes displayed by HL^3^ and H_2_diol in **1**.

The Mn^II^ ions define the four apices of the tetrahedron while the six Mn^III^ ions lie along each edge, therefore describing a trigonal antiprism. The metal ions are connected via four μ_4_-O^2−^ ions to give a [Mn^III^_6_Mn^II^_4_O_4_]^18+^ core such that the supertetrahedron can be thought of as being built from four vertex-sharing [Mn^III^_3_Mn^II^O]^9+^ tetrahedra. Each chloride ion caps one face of the tetrahedron acting as a μ_3_-bridge for the Mn^III^ ions (Mn-Br, ∼2.7 Å), while all L^1−^ ligands display the same coordination mode, chelating to each Mn^II^ ion and bridging between two different Mn^III^ ions (Scheme 1).

The two protonated HL ligands complete the coordination sphere of Mn10 (and s. e.), by terminally bonding to it through the N-atom. The metal ions are all six-coordinate and in distorted {MnO_4_N_2_} and {MnO_4_Cl_2_} octahedral geometries for the Mn^II^ and Mn^III^ ions, respectively. The only exceptions are Mn8 and Mn10, that are hepta-coordinated and in distorted {MnO_5_N_2_} and {MnO_4_N_3_} pentagonal bipyramidal geometries, respectively.

**FIGURE 3:**
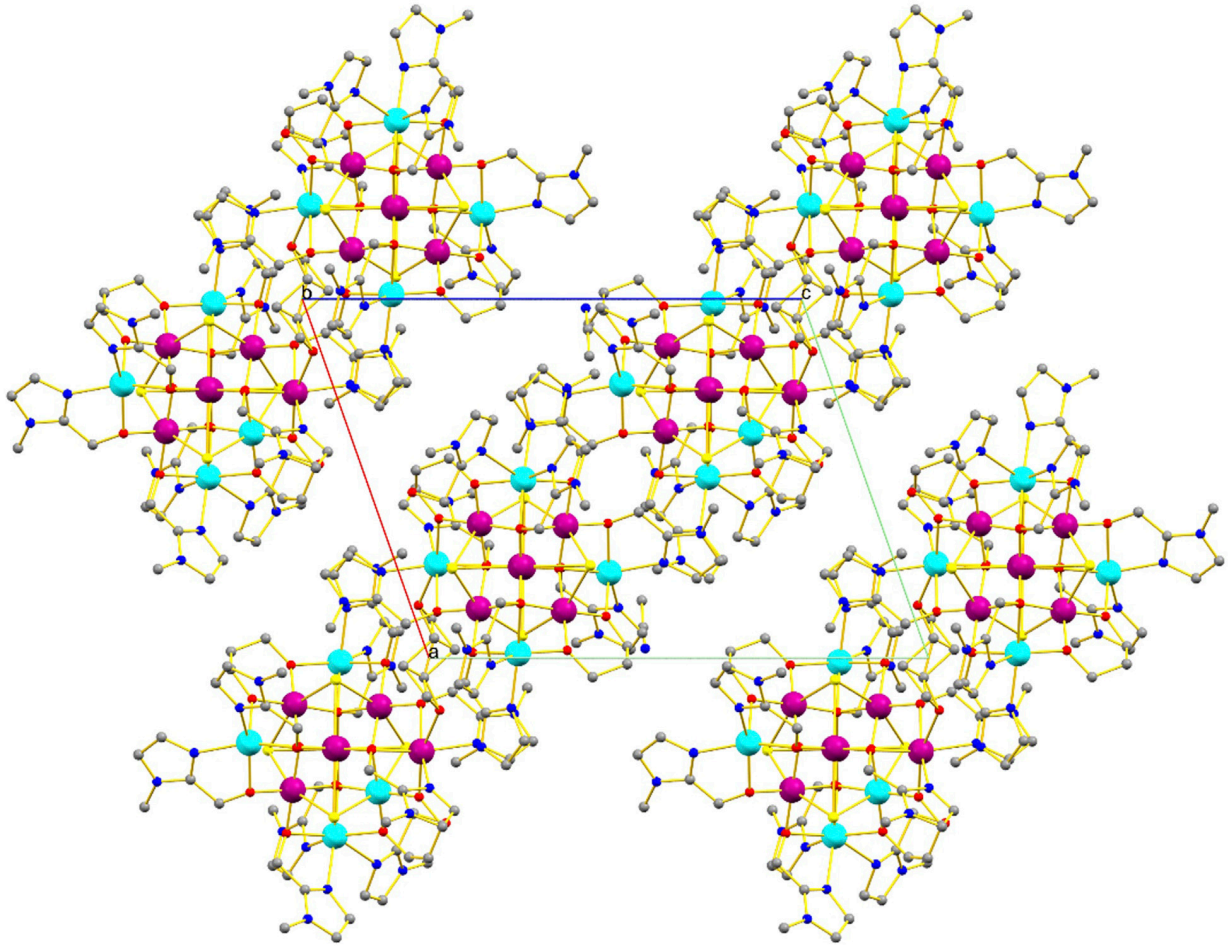
Packing of the clusters of **1** in the extended structure viewed down the *b*-axis of the unit cell. H-bonds are highlighted with thin black dotted lines. Color code: Mn^III^ = purple, Mn^II^ = cyan, O = red, N = blue, C = grey. Solvent molecules and H atoms omitted for clarity.

The pd^2−^ ligands chelate Mn3 and Mn9 (and s. e.) and bridge between two different Mn^II^ ions (Mn1/Mn6 and Mn8/Mn10, respectively), completing their coordination sphere. The Mn^III^ ions are Jahn-Teller distorted with the JT axes being defined by the {Cl-Mn-Cl} vectors. Charge balance is maintained by the presence of the two [Mn^II^Cl_4_]^2−^ counter anions. The protonated pdH_2_ ligands form intramolecular H-bonds to the acetone molecules of crystallization (O(H)⋯O, ∼2.9 Å), and the clusters pack in a brickwork like fashion in the extended structure (Figure 3).

**FIGURE 4:**
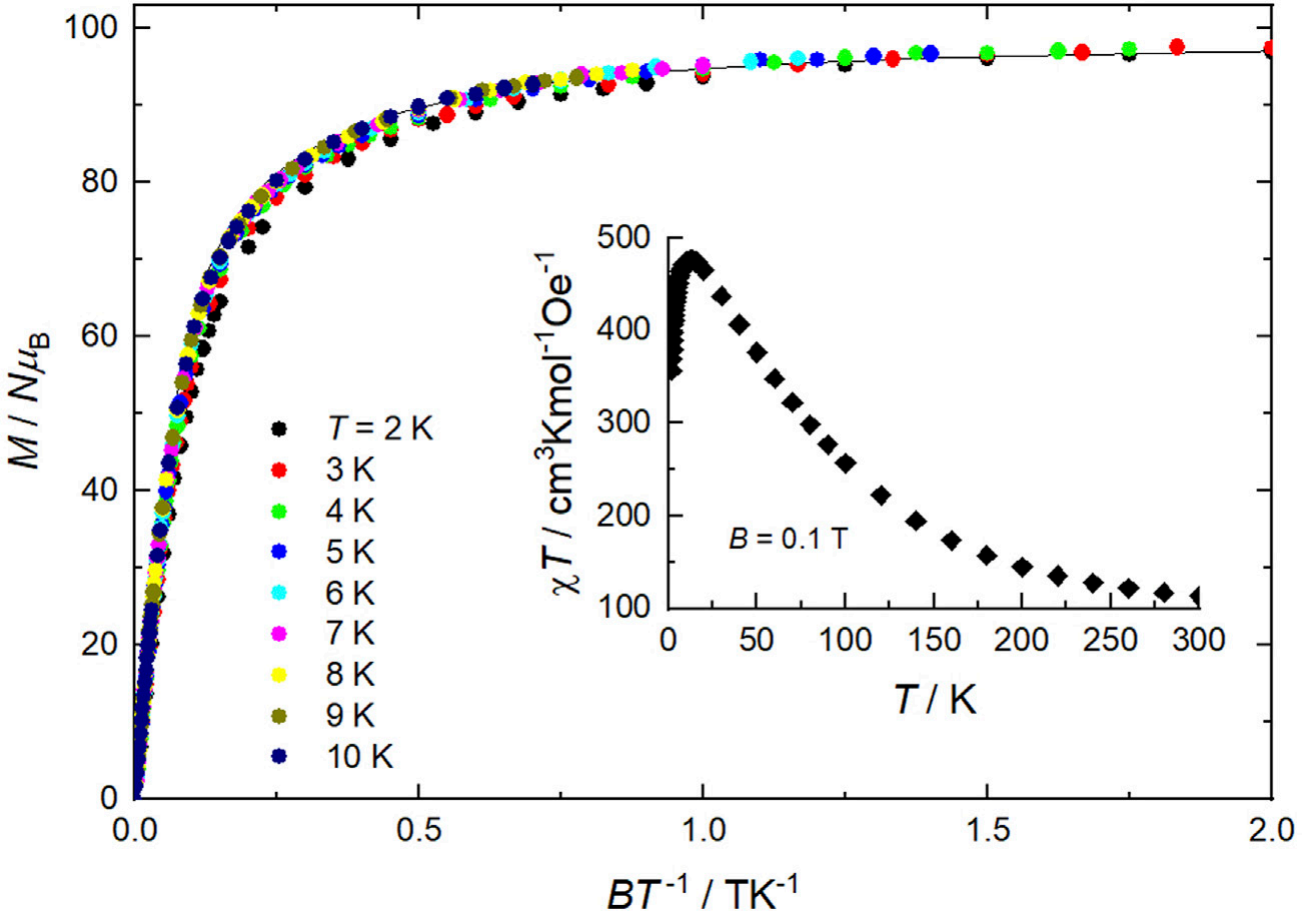
Isothermal low-temperature magnetization (*M*) curves versus *B*/*T* in the ranges *T* = 2–10 K and *B* = 0–7 T, and temperature dependence of the DC magnetic susceptibility at *B* = 0.1 T (as *χΤ*, inset) for **1**. The solid line is the fit of the *M* data, see main text.

Direct current (DC) magnetic susceptibility (*χ*) data on a sample of **1** were collected in the 2–300 K temperature range in an applied magnetic field of *B* = 0.1 T, and are plotted as *χT* versus *T* in Figure 4 (inset). The experimental *χT* value at room temperature (113.1 cm^3^ K mol^−1^) is higher than the value expected for 10 Mn^II^ and 12 Mn^III^ noninteracting ions per formula unit (79.75 cm^3^ K mol^−1^). More importantly, *χT* steadily increases on lowering the temperature, reaching a maximum of 476.4 cm^3^ K mol^−1^ at 12.5 K, before dropping down to 356.8 cm^3^ K mol^−1^ at 2.0 K. The magnetic behavior of **1** denotes predominant ferromagnetic interactions, likely associated with a *s* = 22 ground state for each [Mn^III^_6_Mn^II^_4_] supertetrahedron in close analogy with previous studies (Manoli et al., 2007). Indeed, assuming that all interactions within each super supertetrahedron are ferromagnetic, the effective spin at low temperatures is the sum of 4 *s* = 5/2 (Mn^II^) and 6 *s* = 2 (Mn^III^) spins, leading to a net *s* = 22. Weaker antiferromagnetic correlations between the supertetrahedra and/or Zeeman effects can account for the low-temperature *χT* decrease. Alternating current (AC) magnetic susceptibility measurements show no out-of-phase signal, hence no single-molecule magnet behavior for **1**, as expected from the high symmetry of the supertetrahedron (Manoli et al., 2007). Isothermal magnetization (*M*) measurements were collected in the ranges 0–7 T and 2–10 K. The *M* data plotted versus *B*/*T* merge nicely into a single curve, except for the slight deviation of the data at the lowest temperature of 2 K (Figure 4). The relatively fast variation at low fields further confirms the predominant ferromagnetic interactions in **1**. The saturation reached at low temperatures corresponds precisely to 98.0 *Nμ*_B_, which is consistent with the value expected from 2 *s* = 22 [Mn^III^_6_Mn^II^_4_] supertetrahedra and 2 *s* = 5/2 [Mn^II^Cl_4_] anions, all for *g* = 2.0 and negligible anisotropies. For *T* > 2 K, the experimental data are well modeled by the sum of the corresponding Brillouin functions (solid line in Figure 4).

**FIGURE 5:**
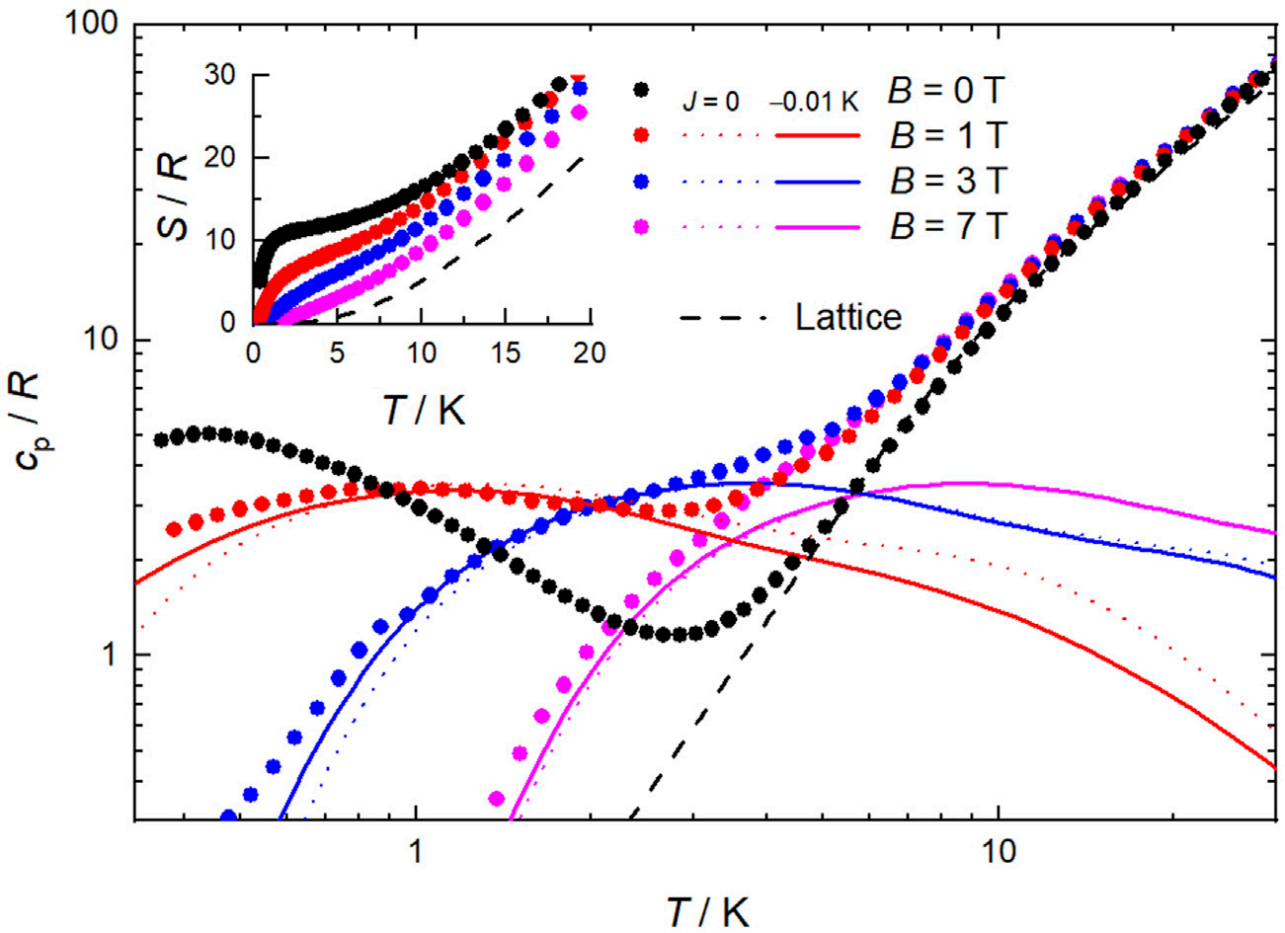
Temperature dependence of the heat capacity (*c*_p_) and entropy (*S*, inset), both normalized to the gas constant, for selected applied field values for **1**. Experimental data = symbols, modelling = lines, including the lattice contribution (dashed line).

Calorimetry experiments were conducted on a pressed pellet sample of **1** for *B* up to 7 T and temperatures between 0.3 and 30 K. At high temperatures, the heat capacity (*c*_p_, Figure 5) is dominated by the nonmagnetic contribution associated with lattice phonon vibrations, which follows Debye’s law (dashed line) that, below ca. 5 K, simplifies to *c*_p_/*R* = *aT* ^3^, where *a* = 2.5 × 10^−2^ K^−3^ and *R* is the gas constant. At low temperatures, *c*_p_ depends strongly on *B*, as expected in the case of ferromagnetic correlations. Note that the heat capacity of an equivalent system with ten Mn^II^ and twelve Mn^III^ ions per formula unit, but without any interaction, would be drastically different from the experimental data. We tentatively modeled the magnetic contribution to *c*_p_ for *B* ≥ 1 T as the sum of Schottky-like anomalies for two noninteracting *s* = 22 [Mn^III^_6_Mn^II^_4_] supertetrahedra, in addition to two noninteracting *s* = 5/2 [Mn^II^Cl_4_] anions, per formula unit, with no anisotropies (dotted lines in Figure 5). While the agreement with the experimental data is acceptable, a better description can be achieved by adding an isotropic (super)exchange magnetic interaction between the two supertetrahedra in the form of a Hamiltonian of type $H=-J\vec{s_{1}}\cdot \vec{s_{2}}$ , where $s_{1}=s_{2}=22$ and J=−0.01 K, denoting a weak but significant antiferromagnetic “intradimer” interaction (solid lines in Figure 5). The anomaly of the zero-field *c*_p_ is seen to increase up to values higher than the other anomalies, suggesting that “interdimer”/intercluster interactions, likely of dipolar origin and thus rather weak, are taking part in the ordering mechanism at such low temperatures below 1 K. From the experimental *c*_p_, we evaluate the temperature and field dependence of the entropy (*S*, inset of Figure 5), according to

$$S=\int_{0}^{T}\frac{c_{p}}{T^{\prime }}dT^{\prime }.$$

The magnetic contribution to *S* at zero-applied field is seen to level off at temperatures between ca. 2–4 K to the value corresponding to 2 *s* = 22 [Mn^III^_6_Mn^II^_4_] supertetrahedra and 2 *s* = 5/2 [Mn^II^Cl_4_] anions, i.e., *S* = 2×[ln(2 × 22 + 1)+ln(2 × 5/2 + 1)]*R* = 11.2*R =* 19.3 Jkg^−1^ K^−1^. At higher temperatures, *S* increases steadily with temperature, mainly because of the nonmagnetic lattice entropy.

**FIGURE 6:**
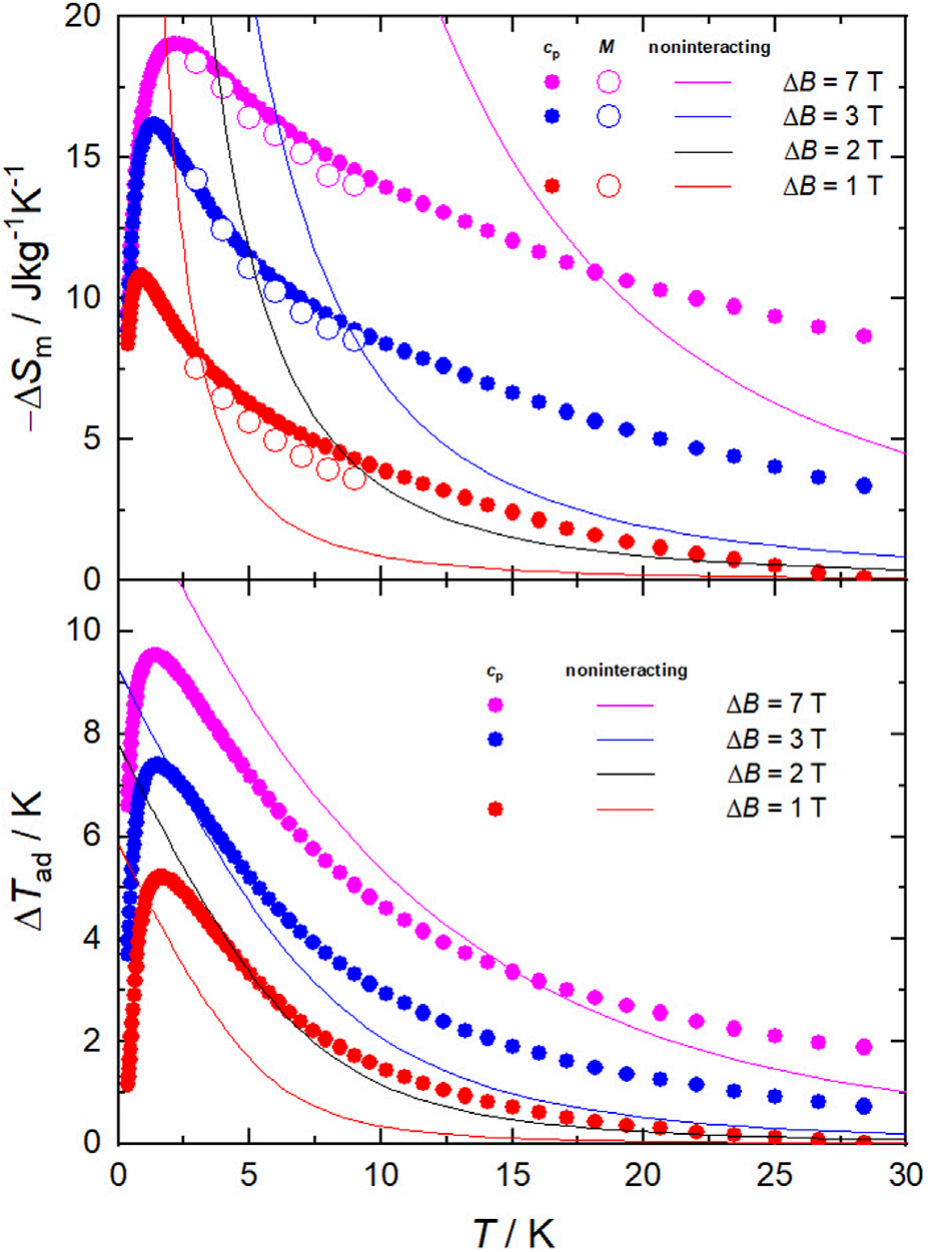
Temperature dependence of the magnetic entropy change (−Δ*S*_m_, top) and adiabatic temperature change (Δ*T*_ad_, bottom) for **1**, obtained from the experimental magnetization (empty symbols) and heat capacity (filled symbols) data. Depicted for comparison are the calculated −Δ*S*_m_ and Δ*T*_ad_ (solid lines), at the indicated Δ*B*, for a hypothetical compound equivalent to **1** but with no magnetic interactions.

Next, we evaluate the magnetocaloric effect, as differences between the entropy curves in Figure 5. The temperature dependencies of Δ*S*_m_ and Δ*T*_ad_ are depicted in Figure 6 for several applied-field changes Δ*B* = (*B*−0), i.e., full demagnetization from *B* = 1, 3 or 7 T. The entropy changes are also computed by applying the Maxwell relation to the magnetization data, namely

$${\left(\frac{\partial S}{\partial B}\right)}_{T}={\left(\frac{\partial M}{\partial T}\right)}_{B},$$

whose results are plotted in Figure 6. As can be seen, both sets of Δ*S*_m_ data, complementarily evaluated from *S* (hence *c*_p_) and *M* data, respectively, agree very well with one another, therefore proving the validity of our approach. The strongest MCE takes place at low temperatures near 2 K. Specifically, the maximal −Δ*S*_m_ = 19.2 Jkg^−1^K^−1^ (which corresponds to the full entropy content for *s* = 22 spins) occurs at *T* = 2.1 K, while the maximal Δ*T*_ad_ = 9.6 K at *T* ≡ *T*_e_ = 1.4 K, both for Δ*B* = 7 T (Figure 6), where *T*_e_ is the ending temperature of the demagnetization process. The observed maximum of −Δ*S*_m_ is relatively large and compares positively with most Mn-based molecular clusters reported for magnetic refrigeration (Table 1), but less favorably with respect to selected Gd-containing molecular compounds (Evangelisti et al., 2011; Sharples et al., 2014; Konieczny et al., 2022; Tziotzi et al., 2023; Zhai et al., 2024). Where **1** excels is in the temperature range over which the entropy change remains fairly large, e.g., −Δ*S*_m_ = 10.5 Jkg^−1^K^−1^ at *T* = 20 K and −Δ*S*_m_ = 8.7 Jkg^−1^ K^−1^ at *T* = 30 K, which are equivalent to 54% and 45% of the observed maximal value, respectively. That is, the strength of the MCE decreases just by half after increasing the temperature by an order of magnitude. This behavior originates from the broad Schottky-like anomaly (Figure 5), which in turn results from the ferromagnetic interactions that are robust to the applied fields. Clusters with a high-spin ground state generated from strong ferromagnetic correlations, such as **1**, are uncommon (Konieczny et al., 2022). These characteristics can be an advantage to MCE, if exploited properly. Clearly, the entropy of a hypothetical molecule having *n* spins *s* is smaller when the spins couple ferromagnetically, yielding a net spin *ns* at low temperature, than when they do not, i.e., ln(*ns*) < *n*×ln(*s*). Therefore, if one targets the largest MCE value, interactions should be avoided. However, the release of the entropy with temperature/field depends on the strength of the ferromagnetic interactions, or their lack thereof, being more abrupt when such interactions are important and hence facilitating a relatively large MCE with small changes of temperature/field (Evangelisti and Brechin, 2010). This behavior is clearly seen in **1**, specifically in the temperature and field dependence of its −Δ*S*_m_ and Δ*T*_ad_ figures of merit that we plot in Figure 6 together with those calculated for a system of ten Mn^II^ and twelve Mn^III^ noninteracting ions per formula unit, for comparison. For any given Δ*B*, larger −Δ*S*_m_ and Δ*T*_ad_ can be produced at the lowest temperatures in the absence of interactions, while **1** outperforms the noninteracting system over a broad temperature/field range. For instance, to reach −Δ*S*_m_ = 4.5 Jkg^−1^K^−1^ at *T* = 8.4 K, or similarly Δ*T*_ad_ = 3.3 K at *T* = 5.0 K, an applied-field change of Δ*B* = 2 T would be needed with the noninteracting system, while just Δ*B* = 1 T would suffice with **1** (Figure 6).

**TABLE 1:** Mn-based molecular clusters proposed for magnetic refrigeration; maximal magnetic entropy changes, −Δ*S*_m_, in units of Jkg^−1^K^−1^ and for Δ*B* = 7 T (or 9 T, where indicated by *); temperatures of maximal −Δ*S*_m_, in K; references.

Mn-based molecular cluster	−Δ*S*_m_	*T*	Reference
[Mn_10_O_4_Br_4_(amp)_6_(ampH_2_)_3_(HampH_2_)]Br_3_ [Mn_10_(OH)_6_(amp)_4_(ampH)_4_I_4_(EtOH)_4_]I_4_·12EtOH [Mn_14_(OH)_2_(Hpeol)_4_(H_2_peol)_6_I_4_(EtOH)_6_]I_4_ {Mn(bpy)_3_}_1.5_[Mn_32_(thme)_16_(bpy)_24_(N_3_)_12_(OAc)_12_](ClO_4_)_11_ [Mn_10_(μ_3_-O)_4_(HL_1_)_6_(μ_3_-N_3_)_3_(μ_3_-Br)(Br)](N_3_)_0.7_(Br)_0.3_·3MeCN·2MeOH [Mn_17_(μ_4_-O)_8_(μ_3_-Cl)_4_(μ,μ_3_-O_2_CMe)_2_(μ,μ-L^2^)_10_Cl_2.34_(O_2_CMe)_0.66_(py)_3_(MeCN)_2_]·7MeCN [Mn_19_(μ_4_-O)8(μ3,η^1^-N_3_)_8_-(HL^3^)_12_(MeCN)_6_]Cl_2_·10MeOH·MeCN [Mn_4_(N_3_)_7.3_Cl_0.7_(4,5-diazafluoren-9-one)_4_] [Mn(glc)_2_(H_2_O)_2_] [Mn(tmphen)_2_]_4_[Nb(CN)_8_]_2_·14H_2_O·7MeOH [(CH_3_)_2_NH_2_]_6_[Mn_38_(m_6_-CO_3_)_9_(m_2_-O)_6_Cl_24_(bmpbt)_12_(H_2_bmpbt)_6_][MnCl_4_]_2_ [Mn(H_2_O)_2_]_6_[Mn_21_(_L_-TartH_−2_)_2_(_L_-TartH_−1_)_10_(μ_2_-O)_6_(μ_4_-O)_8_](H_2_O)_11_ [Mn_20_O_8_(L)_16_(HL)_2_(pd)_4_(pdH_2_)Cl_8_][MnCl_4_]_2_·3MeCN·9C_2_H_6_O	13.0 17.0 25.0 18.2 10.3* 13.3* 8.9* 19.3 60.3 8.3 14.5 8.8 19.2	2.2 5.2 3.8 1.6 2.6 5.2 4.2 4 1.8 2.0 2.0 5.0 2.1	Manoli et al. (2007) Manoli et al. (2008) Manoli et al. (2008) Evangelisti et al. (2009) Nayak et al. (2010) Nayak et al. (2010) Nayak et al. (2010) Zhao et al. (2013) Chen et al. (2014) Arczyński et al. (2017) Wu et al. (2021) Xu and Xu (2022) This work

Finally, we compare the magnetic and magnetocaloric properties of **1** with those of the structurally related cluster [Mn_10_O_4_Br_4_(amp)_6_(ampH_2_)_3_(HampH_2_)]Br_3_ (Manoli et al., 2007). Magnetically, both compounds behave similarly owing to the *s*=22 ground state of the ferromagnetic and isotropic [Mn^III^_6_Mn^II^_4_] supertetrahedra. The “dimer” coupling in **1** is relatively weak ( J=−0.01 K) and marginally affects the properties at the lower temperatures. A further small difference comes from the presence of the noninteracting [Mn^II^Cl_4_] anions in **1**, which add a *s* = 5/2 paramagnetic contribution for every [Mn^III^_6_Mn^II^_4_] supertetrahedron. Given the close similarities from the magnetic standpoint, one would expect an equally similar MCE. However, their entropy changes differ drastically with respect to one another, e.g., the maximal values are −Δ*S*_m_ = 19.2 and 13.0 Jkg^−1^K^−1^ for **1** and [Mn_10_O_4_Br_4_(amp)_6_(ampH_2_)_3_(HampH_2_)]Br_3_, respectively, both for Δ*B* = 7 T and at ca. the same temperature (2.1 vs. 2.2 K, respectively). Such a difference is almost entirely ascribed to their molecular weights, namely 4,831.09 vs. 2,902.37 g/mol, respectively, which implies a significantly higher magnetic density in **1**, which comprises two [Mn^III^_6_Mn^II^_4_] supertetrahedra and two [Mn^II^Cl_4_] counter anions per formula unit. This apparent contradiction is resolved by reporting −Δ*S*_m_ in molar units, e.g., the maximal values reached by both compounds correspond to their full available entropy contents for *s* = 22 spins.

## 4 Concluding remarks

We have synthesized a molecular magnetic refrigerant, characterized by a mixed-valence [Mn_20_] cationic cluster and two [Mn^II^Cl_4_] counter anions. Each [Mn_20_] unit can be magnetically described as formed by two ferromagnetic and isotropic [Mn^III^_6_Mn^II^_4_] supertetrahedra with *s*=22, coupled mutually by a weak antiferromagnetic interaction. Such a large spin ground state promotes an enhanced magnetocaloric response to the applied magnetic field and a MCE that remains relatively large over a broad temperature range, e.g., from −Δ*S*_m_ = 19.2 Jkg^−1^K^−1^ at 2.1 K to −Δ*S*_m_ = 8.7 Jkg^−1^K^−1^ at *T* = 30 K, for Δ*B* = 7 T, that is roughly a decrease by a factor of 2 upon increasing the temperature by an order of magnitude. Complex **1** differs from the closely related and previously reported cluster [Mn_10_O_4_Br_4_(amp)_6_(ampH_2_)_3_(HampH_2_)]Br_3_ (Manoli et al., 2007) because **1** is significant lighter, that is, its larger magnetic density makes it a better magnetic refrigerant.

## Data Availability

The raw data supporting the conclusions of this article will be made available by the authors, without undue reservation.
